# Hip Fractures in Malta: Does Delay in Surgery Affect Clinical Outcomes?

**DOI:** 10.7759/cureus.75467

**Published:** 2024-12-10

**Authors:** Kyle Muscat

**Affiliations:** 1 Surgery, Mater Dei Hospital, Msida, MLT

**Keywords:** covid 19, fragility hip fractures, length of hospital stay (los), mortality rate, revision surgery, surgical delay

## Abstract

Introduction: Hip fractures are common and are a major cause of significant morbidity and mortality in the elderly population, particularly when treatment is delayed. The British Orthopaedic Association’s (BOA) guidelines state that surgical treatment should be performed within 36 hours of admission. This study aimed to investigate the effects of delays in surgery on clinical outcomes and to evaluate mortality rates over a three-year follow-up period following proximal femoral fractures.

Methodology: This was a single-center, retrospective observational study of all patients aged ≥60 years admitted with low-energy hip fractures between June 1, 2020, and November 30, 2020. A total of 205 patients were included and followed up for three years. Data were collected from electronic medical records and operating theater notes. Statistical analysis was performed to analyze the effects of delay in surgery on clinical outcomes.

Results: A 45.9% all-cause mortality rate was observed at three years post-hip fracture in this study. A delay of more than 36 hours to surgery was associated with a statistically significant increase in both length of hospital stay and mortality at one and three years, while no difference was observed in hip-related complications.

Conclusions: The three-year mortality rate compares well with those found in the literature. A delay in the surgical management of hip fractures is associated with overall worse clinical outcomes, with a higher mortality rate at three years.

## Introduction

The global prevalence of osteoporotic hip fractures is on the rise due to an increasing life expectancy and the resultant aging population. Proximal femoral fractures account for up to 40% of fragility fractures [1] and are a major cause of long-term morbidity and mortality. The reported one-year mortality rate following a hip fracture in the literature ranges from 20% to 35%, with a three-year mortality rate of 48.8% [2,3].

The British Orthopaedic Association’s (BOA) Standard guidelines state that surgical treatment in frail patients should be performed within 36 hours of admission [4]. There is contrasting literature regarding the effects of delayed surgery on mortality. Several studies have shown a decreased mortality rate, complication rate, and hospital stay in patients undergoing early surgery [5,6]. However, several other studies have concluded that a delay in surgery did not affect mortality [7,8]. A 2011 literature review by Simunovic et al. noted contrasting evidence regarding the effects of surgical delay on mortality, postoperative complications, and length of hospital stay [9]. The main advantage of early surgery is minimizing the time a patient is confined to bed rest, thereby reducing complications such as pressure sores, deep vein thrombosis, and urinary tract infections. On the other hand, those advocating delayed surgery argued that this allowed for better preoperative optimization of patients to prevent perioperative complications [9].

The aim of this study was to evaluate the mortality rates over a three-year follow-up period following a hip fracture and investigate the effects of surgical delay on clinical outcomes and mortality rates. The study also investigated any possible association between operation type and mortality.

## Materials and methods

A single-center, retrospective observational study of all patients admitted with hip fractures during six months, between June 1, 2020, to November 30, 2020, at Mater Dei Hospital, Msida, Malta. They were identified using the hospital’s trauma operations registry by filtering AO/Orthopaedic Trauma Association (OTA) fracture types 31A-31B [10] and then visualizing the admission radiographs.

All patients older than 60 years admitted with a low-energy proximal femoral fracture (AO/OTA fracture types 31A and 31B) with complete medical records and radiographs were included in this study. A total of 205 patients were included in the cohort. Exclusion criteria for this study included patients with pathological fractures, re-fractures, polytrauma (or an Injury Severity Score [ISS] > 15), and incomplete medical records (Table 1).

**Table 1:** Inclusion and exclusion criteria. AO/OTA, AO Foundation/Orthopedic Trauma Association; ISS, Injury Severity Score

Inclusion criteria	Exclusion criteria
Age > 60 years	Pathological fractures
Low-energy trauma (fall from own height)	Re-fractures
AO/OTA fracture types 31A-31B	Polytrauma (or ISS > 15)
	Incomplete medical records

Routinely collected data were used for the study. Data on patient demographics, mechanism of injury, American Society of Anesthesiologists (ASA) grade, hip fracture type, operation type, time to surgery, length of hospital stay, re-presentations with hip-related problems, and mortality were collected from the hospital’s electronic medical records, namely, iSoft Clinical Manager (hospital’s main software), Electronic Case Summary (hospital’s discharge database), and Picture Archiving and Communication System (hospital’s imaging database). Data were collected for up to three years post-fracture for each patient. 

The outcomes measured were mortality rates at three months, one year, and three years; length of hospital stay; re-presentation with hip-related problems (avascular necrosis of femoral head, malunion, nonunion, deformity, metalwork failure, peri-prosthetic fracture, dislocation, and heterotrophic ossification); and revision surgery.

Data collection and compilation were done using Microsoft Excel (Microsoft Corporation). Descriptive statistics were used to calculate the mean and standard deviation (±SD). Categorical variables were reported as frequency (*n*) and percentage. Statistical analyses were performed using the Pearson chi-squared test or Fisher’s exact test to compare categorical variables, and the Student’s t-test was used to compare means. A *P*-value of ≤0.05 was considered statistically significant.

## Results

**Figure 1:**
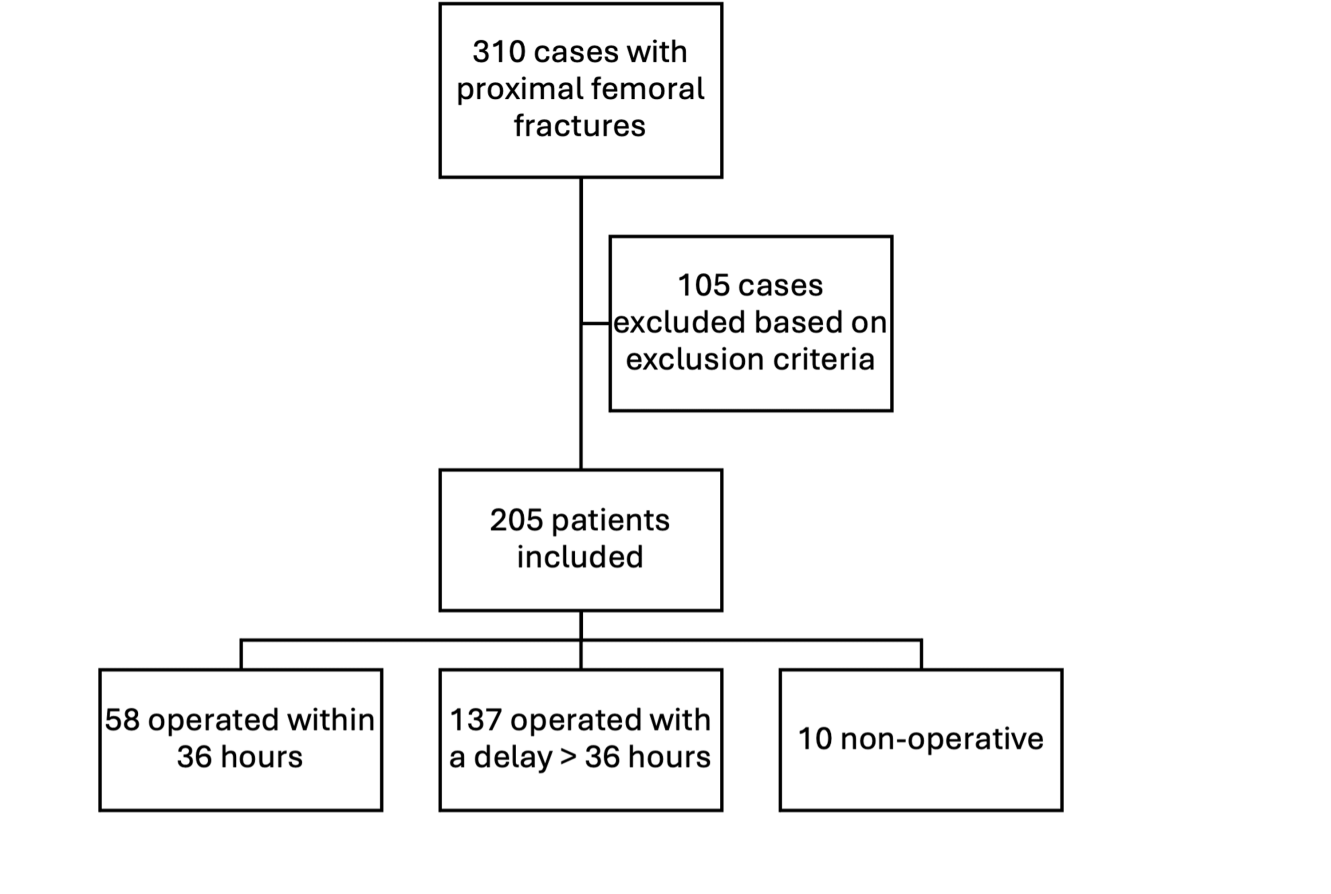
Flowchart of the study population selection process.

Between June 1, 2020, and November 30, 2020, 310 patients with proximal femoral fractures were admitted to Mater Dei Hospital in Msida, Malta, and entered into the hospital’s trauma registry system. After applying the inclusion and exclusion criteria, a total of 205 patients were identified and included in this study (Figure 1). 

There was a clear female predominance with 66.8% (*n* = 137) of the study population, with a mean age of 80.31 ± 8.24 years. The male group had a mean age of 80.76 ± 8.57 years and a mortality rate of 54.4%, compared to a 41.6% mortality rate observed in the female group. This was not found to be statistically significant (*P* = 0.102). 

The overall mortality rate at the end of the three-year follow-up period was found to be 45.9% (*n* = 94). The three-month and one-year observed mortality rates were 17.6% (*n* = 36) and 32.2% (*n* = 66), respectively (Table 2).

**Table 2:** Basic demographics and overall outcomes. SD, standard deviation

Variable	*n *(%)
Number of patients	205
Age (years, mean ± SD)	80.46 ± 8.33
Male	68
Age (years, mean ± SD)	80.76 ± 8.57
Mortality	37 (54.4%)
Female	137
Age (years, mean ± SD)	80.31 ± 8.24
Mortality	57 (41.6%)
Time to surgery (days, mean ± SD)	4.36 ± 3.13
Length of hospital stay (days, mean ± SD)	20.49 ± 15.23
Mortality	
3 months	36 (17.6%)
1 year	66 (32.2%)
3 years	94 (45.9%)

A total of 195 patients underwent operative management, while the remaining 10 patients were managed non-operatively. The most common operative interventions were fixation, with a dynamic hip screw (DHS) or proximal femoral nail (PFN), and replacement using a total hip replacement (THR) or hemiarthroplasty (Hemi). The THR procedure was the only one that showed a significantly lower three-year mortality rate when compared with the other operative interventions (*P* = 0.001) (Table 3), whereas none of the patients managed non-operatively survived beyond three years (*n* = 10). 

**Table 3:** Operative procedure and outcomes. DHS, dynamic hip screw; PFN, proximal femoral nail; Hemi, hemiarthroplasty; THR, total hip replacement

Operative procedure	DHS	PFN	Hemi	THR	Non-operative
n	87	35	49	16	10
Three-year mortality, *n* (%)	37 (42.5%)	17 (48.6%)	26 (53.1%)	1 (6.25%)	10 (100%)
Length of stay (days)	21.5 ± 15.5	22.9 ± 11.9	20.7 ± 15.0	10.8 ± 5.5	15.3 ± 13.3
Hip-related problems (revision surgery)	7 (2)	1 (0)	4 (0)	2 (2)	/

Fifty-eight patients (29.7%) were operated within 36 hours of admission, with a mean age of 80.40 ± 8.01 years, while 137 patients had a delay of longer than 36 hours from admission to surgical intervention, with a mean age of 80.15 ± 8.08 years. There was no statistically significant difference between the two groups in terms of mean age; however, a significantly (*P* = 0.003) larger proportion of those operated within 36 hours were graded as ASA 1-2 (47, 81%) as opposed to those who experienced a longer delay to surgery (80, 58.4%).

While there was no difference in three-month mortality rates between the two groups (*P* = 0.11), a significant difference in mortality rates at one and three years following index fracture was noted with *P*-values of 0.04 and 0.027, respectively (Table 4). Those patients operated within 36 hours of admission were also shown to have a significantly shorter length of hospital stay (*P* = 0.02), with a mean stay of 17.8 ± 12.5 days. There was no significant difference between the two groups in terms of hip-related re-presentations and revision surgery. A total of 14 patients (7.2%) re-presented, of whom 4 (2.1%) required revision surgery.

**Table 4:** Surgical delay and outcomes. SD, standard deviation; ASA, American Society of Anesthesiologists; *t*, *t*-value; Chi, Chi-square value

Delay to surgery (n)	<36 hours (58)	>36 hours (137)	*P*-value
Age (years, mean ± SD)	80.40 ± 8.01	80.15 ± 8.08	0.85 (*t* = 0.20)
ASA grade			0.003 (Chi = 8.872)
1-2	47 (81.0%)	80 (58.4%)	
3-4	11 (19.0%)	56 (40.9%)	
Mortality			
3 months	5 (8.6%)	24 (17.5%)	0.11 (Chi = 2.548)
1 year	11 (19.0%)	46 (33.6%)	0.04 (Chi = 4.205)
3 years	18 (31.0%)	66 (48.2%)	0.027 (Chi = 4.882)
Length of stay (days, mean ± SD)	17.8 ± 12.5	22.2 ± 16.2	0.02
Hip-related problems	5 (8.6%)	9 (6.6%)	0.762 (Chi = 0.257)
Revision surgery	1 (1.7%)	3 (2.2%)	1.00 (Chi = 0.044)

## Discussion

It is well known that hip fractures are associated with significant mortality during their in-patient stay as well as following discharge. A three-month mortality rate of 17.6% was found in this study, which is identical to that in a recent study by Postler et al. looking at hip fractures at a level 1 trauma center [11]. Both the one-year and three-year mortality rates, found to be 32.2% and 45.9%, respectively, also compare well with those reported in other studies (20%-35% and 48.8%) [3,12].

In this study, it was noted that patients undergoing a THR had a significantly lower mortality risk at three years when compared to other operative interventions (*P* = 0.001). This is likely due to other confounding factors, as patients undergoing THR are generally healthier and better candidates for surgery. This is reflected in the National Institute for Health and Care Excellence (NICE) guidelines for the management of hip fractures, which recommend considering THR for patients with a displaced intracapsular hip fracture, provided they can walk independently with no more than the aid of a stick, are medically fit for anesthesia and procedure, and are expected to perform activities of daily living independently for at least two years [13]. All patients who did not meet the aforementioned criteria are instead offered a hemiarthroplasty. On further analyzing this subgroup of patients, it was noted that 15 of the 16 patients (93.75%) had an ASA grade of ≤2 in contrast to 115 of 189 (60.85%) in the rest of the study population. There was no difference in mortality between Hemi, DHS, or PFN procedures as has been demonstrated in several studies [14,15].

The BOA currently recommends that all frail patients be operated within 36 hours of admission [4]. In this study, a mean delay of 4.36 ± 3.13 days to surgery was observed, with only 29.7% of patients undergoing operative management within 36 hours of admission. This delay may have been partially influenced by factors related to the COVID-19 pandemic at the time. It is well known that the pandemic placed an increased burden on medical healthcare systems [16], resulting in reduced anesthetic and surgical staff available for theater due to increased critical care demands. Studies have shown that the COVID-19 pandemic resulted in significant delays to surgery for patients suffering a hip fracture [17,18]. However, the 29.7% observed in this study was significantly lower than the 69% reported across the United Kingdom during a similar period in 2020 amid the COVID-19 pandemic [16].

Many previous studies have shown that a greater delay in surgery is associated with increased mortality, including a large systematic review and meta-analysis by Shiga et al. [5,19]. This was also demonstrated in this study, where a delay in surgery of more than 36 hours resulted in higher mortality rates at both one and three years post-fracture, as well as an extended hospital stay. It is also true that these patients were more co-morbid preoperatively, with up to 40.9% classified as ASA grade 3 or higher. Therefore, worse outcomes may have been expected. These patients require further medical optimization before surgery, which also contributes to their delay in surgery. No difference was found between the two groups in terms of re-presentations with hip-related problems and revision surgery cases; however, a low number of such cases were identified during this study.

The limitations of the study were acknowledged. First, although admissions over six months were considered, the final population was still relatively small, as evidenced above. The study was a retrospective, observational study, so there was no control over the two cohorts, resulting in heterogeneity between the cohorts as evidenced by the differences in ASA grades. Finally, electronic medical records were used for data collection, which introduces several limitations in itself such as incomplete data and lack of standardization of inputted data.

## Conclusions

To conclude, the mortality rate three years after a hip fracture in Malta, as observed in this study, was found to be 45.9%. A delay in surgery of more than 36 hours was associated with a significant increase in mortality at both one and three years post-hip fracture, as well as an extended hospital stay. These findings support the BOA's recommendations. Furthermore, THR was associated with a decreased risk of mortality.
